# Response to an unusual outbreak in a high-risk situation

**DOI:** 10.11604/pamj.supp.2017.27.1.12566

**Published:** 2017-05-28

**Authors:** Mahmood Muazu Dalhat, Olufunmilayo Ibitola Fawole, Patrick Mboya Nguku, Meeyoung Mattie Park, Casey Daniel Hall, Nasir Sani-Gwarzo

**Affiliations:** 1 Nigeria Field Epidemiology Training Program, Nigeria; 2 College of Medicine, University Ibadan, Ibadan, Nigeria; 3 Rollins School of Public Health, Emory University; 4 Department of Public Health, Federal Ministry of Health, Abuja, Nigeria

**Keywords:** Public health, epidemiology, outbreak, lead poisoning, disaster management

## Abstract

In 2010, a series of lead poisoning outbreaks linked to artisanal gold processing killed at least 400 young children in Zamfara State in northwestern Nigeria. There were several efforts to respond to the outbreaks as they occurred. Subsequent recurrence of lead poisoning outbreaks within Zamfara and beyond suggested that there were no efforts to mitigate the outbreaks as recommended for disaster management. This case study, to be completed within 3 hours, is suitable for senior level public health officials and those training for such positions. It enables participants to review and apply epidemiological principles for managing disasters and suggest steps toward development of policy recommendations based on the context of environmental lead exposure. It will serve as a generic training module for managers/responders of other natural (floods, heat stroke) and man-made disasters (civil strife, conflict, insurgency) based on the general/standard principle of the complete disaster management cycle.

## How to use this case study

**General instructions:** this case study is designed for training 15-20 participants in a classroom setting. A facilitator leads the participants through the case study as participants take turns reading one or two paragraphs out loud and provides guidance as participants go through each discussion or exercise question. Instructor’s notes are included with each question in the instructor’s version of this case study with suggestions for facilitation and with background materials.

**Audience:** senior level public health officials, public health officials responsible for policy and management, students of public health and/or field epidemiology, and practising (field) epidemiologists.

**Prerequisites:** before using this case study, case study participants should have received lectures or instruction in disaster management

**Materials needed:** Whiteboard or flip chart, markers

**Level of training and associated public health activity:** intermediate - disaster management.

**Time required:** 2-3 hours

**Language:** English

## Case study material

- Download the case study student guide (PDF - 2.86 MB)
- Request the case study facilitator guide
